# Health Selectivity and Rural-Urban Migration in China: A Nationwide Multiple Cross-Sectional Study in 2012, 2014, 2016

**DOI:** 10.3390/ijerph16091596

**Published:** 2019-05-07

**Authors:** Yao Yi, Yu Liao, Lingling Zheng, Mengjie Li, Jing Gu, Chun Hao, Yuantao Hao

**Affiliations:** 1 Department of Medical Statistics and Epidemiology, School of Public Health, Sun Yat-sen University, 74 Zhong Shan 2nd Road, Guangzhou 510080, China; yiyao@mail2.sysu.edu.cn (Y.Y.); liaoy43@mail2.sysu.edu.cn (Y.L.); zhengll8@mail2.sysu.edu.cn (L.Z.); limengjie@mail.sysu.edu.cn (M.L.); gujing5@mail.sysu.edu.cn (J.G.); haochun@mail.sysu.edu.cn (C.H.); 2 Guangdong Provincial Center for Disease Control and Prevention, Guangzhou 511430, China; 3 Guangdong Key Laboratory of Health Informatics, Sun Yat-sen Global Health Institute, Sun Yat-sen University, 74 Zhong Shan 2nd Road, Guangzhou 510080, China

**Keywords:** health selectivity, healthy migrant, salmon bias, rural-urban migration

## Abstract

*Background*: China is undergoing an unprecedented rural-urban migration, which may deeply influence the health of internal migrants. Previous studies suggested that migrants are a selectively healthier population. This paper examines the evidence for and the changes of health selectivity among Chinese internal migrants. *Methods*: We use data from the China Labor-force Dynamics Survey (CLDS), a nationally representative survey conducted in 2012, 2014, and 2016, respectively. The health statuses of four groups of research subjects (out-migrants, returned migrants, rural residents, and urban residents) are measured by general health, physical health, and emotional health. *Results*: By comparing the health status of migrants with that of rural residents, we find supportive evidence for the Healthy Migrant Hypothesis that migrants exhibit better health than rural residents in their hometown. We also add strength to the Salmon Bias Hypothesis that migrants returning to their hometowns are less healthy than those still being outside. However, migrants present worse emotional health in both comparisons. The general and physical health gaps between migrants, rural residents, and returnees widened in all three rounds of the survey, which implies a possibly increasing trend of health selectivity. This study also suggests that bringing family to the destination requires better general and physical health, but not emotional health. *Conclusions*: Migrants are positively selected on general and physical health. The health selectivity in 2012–2016 is highly likely to increase, which means that there are increasing number of obstacles for migrants to overcome. Family migration’s high requirement for health might also contribute to it. It is urgent to establish and improve primary health care service systems in rural areas in current circumstances.

## 1. Introduction

Studies on the relationship between immigration to the United States and health status have revealed a “Hispanic Health Paradox:” although Hispanic immigrants have relatively low socioeconomic status and less access to health care and resources, their health status is better than non-migrant counterparts in their home countries and in the U.S. [1,2,3]. This phenomenon has been discovered in immigration studies in Canada [4], Australia [5], and various European countries [6]. Similar findings have been reported in internal rural-urban migration in developing countries [7,8,9]. Migrants represent a selectively healthier population as indicated by birthweight, mortality rates [10], and adult health status, though the health advantage tends to dissipate over time because of acculturation [11,12] and downward assimilation in health [13,14,15].

There are two self-selection processes regarding the health of migrants [16]. The first one is known as “Healthy Migrant Hypothesis.” This hypothesis holds that migrants exhibit better health than non-migrants in their hometown because they need better health to overcome foreseeable obstacles in the migration and settlement processes. The second one is called “Salmon Bias Hypothesis,” which posits that migrants returning to their hometown are less healthy than those still being away from homes [17,18]. The selective return migration, if ignored, may upwardly bias the immigrants’ health status. These two self-selection processes explain why migrants appear to be healthier than residents. The Healthy Migrant Hypothesis has been bolstered in numerous studies, while fewer studies have lent support to the Salmon Bias Hypothesis. The main difficulty for the latter lies in tracing international migrants, since once they have left the host country, it is almost impossible to collect data from their home countries. In contrast, the study of internal migration allows to follow individuals both in and out of migrant destinations, which would help to better evaluate the selective return process.

China has the largest internal migrant population in the world, reaching 245 million by the end of 2016 [19]. The majority of them are rural-to-urban migrants [20]. Generally speaking, the rural-urban migration is distinct from urban-urban migration with regard to the causes and settlement. In China, the household registration system (hukou) separates citizens with rural versus urban hukou. Rural migrants have limited rights to employment in public sectors, schooling, medical insurance, and public assistance programs in cities [21,22]. Thusly, it is necessary to understand the health differences in terms of both hukou status and migrant status. 

Studies on migrants’ health have yielded inconsistent conclusions. International experience provides hints on both physical and psychological health advantages of immigrants [23]. A study of Thailand found that internal migrants were physically healthy but mentally ill [24]. Research on China’s internal migrants reported inconsistent results depending on different migrant groups and different psychological health indicators [25,26]. Migrants had more psychological distress than urban residents [27], but they had fewer depression risks than rural residents [28]. Nevertheless, there has been little literature on the psychological or emotional health of both returned migrants and out-migrants.

Furthermore, evidence has shown that the intensity of health selection might change over time. Previous studies in Mexico and the U.S. found that smoking selectivity increased among women because of changes in Mexico’s tobacco control policies [29]. Another study found increased health selection of obesity as the Mexican obesity prevalence increased [30]. Research in China revealed that the impact of health on out-migration became less intense over time [31]. This might be due to the increasing migrant networks following the relaxation of particular hukou provisions. 

Health selectivity might be affected by both characteristics of migrants and the policy environment in China. From the perspective of the migrants themselves, family migrants might change their decision to migrate or return. The migration of parents and children as a whole, rather than individual adults alone, has prevailed in China in recent years. Family support from former movers helps the new-arrivals to find jobs and establish social connections in a new place [32], which may lead to reduced health selectivity. However, the migrant children and old people have a higher demand for social resources, which is hard to satisfy and may affect the self-selection of migrants. Research in Indonesia found more psychological costs for migrants moving alone due to family separation and lessened social support [8]. Yet, whether family migration is positive or negative on health is unclear in China, as are its impacts on health selectivity. 

From the external policy perspective, claiming benefits from the New Rural Cooperative Medical System for migrants working away from their hukou locations is still limited [33,34]. The Chinese government has gradually integrated migrants into a unified health system since 2009. We speculate the improvements of social services and diminishing stringent requirements for migrants might reduce health-related return migration, thus further the intensity of health selectivity. Previous studies as mentioned above only examined health selective out-migration and found decreased health selectivity in 1997–2009 [31]. Hitherto, no previous studies reported the changes of health selective out-migration and return-migration simultaneously in China’s context. 

This study aims to explore these changes of health selectivity using data from the China Labor-force Dynamics Survey (CLDS), a nationally representative survey. The two immigrant health selectivity hypotheses are to be examined utilizing comparisons between out-migrants, returned migrants, rural residents, and urban residents, controlling for factors that affect health. We will examine the change of health selectivity in 2012–2016 and further investigate the implication of health selectivity in family migration.

## 2. Materials and Methods 

### 2.1. Data and Analytic Sample

Data used in this study are from the 2012, 2014, and 2016 rounds of the China Labor-force Dynamics Survey (CLDS). The CLDS is a nationally representative, longitudinal survey conducted by Sun Yat-sen University (http://isg.sysu.edu.cn/node/353). It adopts a multi-stage stratified sampling method, covering 29 provinces in China (except for Hong Kong, Macao, Taiwan, Hainan, and Tibet). The study is conducted in accordance with the Declaration of Helsinki, and the protocol is approved by the Institutional Review Board of Sun Yat-sen University. More information on the design, sampling procedures, and methodology is documented elsewhere [35,36,37]. 

The CLDS initiatively adopts a rotation group design that can better adapt to China’s drastic social change and be more cross-sectionally representative in each round. The first round was implemented in 2012 and followed up every two years. Data were collected from individuals, families in the remaining communities, and new communities in a new rotation group. With the rotation group design, new study participants from the same county were added to the sample over time to make the dataset cross-sectionally representative.

CLDS survey interviewed 16,253, 23,594, and 21,086 individuals in 2012, 2014, and 2016, respectively. A total of 51,530 pooled individual cases were included in our analytic sample, with 3004 excluded due to their being younger than 15 or older than 64 years of age. We also excluded 3643 people who were not in the four categories (defined below) and 2756 cases with missing data in key variables. 

We categorized the sample individuals into four types: Out-migrants, returned migrants, urban residents, and rural residents. Out-migrants were defined as rural-hukou individuals who had left their registered residence (according to the Chinese household registration system “hukou”) for six months or longer at the time of the survey. Returned-migrants were defined as rural-hukou individuals who had left their registered residence for more than six months but returned now with no plans to depart again soon. Migrants temporarily returning for holidays, weddings, or building new homes were excluded. People without migration experience were divided into urban residents (with urban hukou) and rural residents (with rural hukou). A few individuals who had changed hukou status within a year of the survey were excluded for such a decision might be endogenously affected by health status.

### 2.2. Measurement

Health status was measured by three self-rated health indicators: General health, physical health, and emotional health. These indicators were directly selected from the 12-item Short Form Health Survey (SF-12) [38,39], and could represent different dimensions of health. For general health status, respondents were asked the question “In general, how would you rate your health?” The answers “excellent/very good/good” and “fair/poor” were regarded to mean good health and poor health, respectively. This transformation was the same as previous studies [36]. For physical health status: “During the past four weeks, did physical health problems affect your work or other regular daily activities?” For emotional health status: “During the past four weeks, did emotional problems (for instance, depression or anxiety) affect your work or other regular daily activities?” For the last two questions, the answers “none/few” and “sometimes/ frequently/always” were regarded to mean good health and poor health respectively. The original answers to each question were on a five-point scale, and they were only used in univariate analysis. We transformed them into dichotomous outcomes as above and used in multivariate analysis to simplify the interpretation of the results. Poor health was coded 0, and good health was coded 1.

The factors which might affect the individual’s health were considered in this study, including variables of human biology, environment, lifestyle, and healthcare organization [40]. Sociodemographic characteristics were included such as gender, age, marital status (“married or cohabitating,” “single, divorced, or widowed”), education level (“primary or lower,” “junior,” “senior,” “college or above”), socioeconomic status (self-rated using the 10-level Likert Scale), living alone or living with family. As for working environmental factors, we evaluated the exposure of occupational hazards, including dust, ionizing radiation, toxic or corrosive chemicals, and physical, biological, or other occupational hazards, during the most recent year in their work environment. Workweek (“≤44h/w,” “>44h/w,” “0h/w”) was used to measure the working status. Working more than 44 hours a week was defined as overtime according to the regulation of China’s labor law. Health behaviors include smoking history (one or more cigarettes per day for a year or more) and drinking history (at least once a week). Health services accessibility included financial accessibility (whether the respondent had health insurance) and geographic accessibility (whether one had fitness facilities in residential community and the numbers of hospitals in residential community).

### 2.3. Statistical Analysis

The software Stata 13 was used in statistical analysis. Chi-square tests or one-way analysis of variance were performed to compare variables between four categories of the population. LSD pairwise comparison was used to compare the health scores of the four categories in univariate analysis. A first set of multiple logistic regressions were performed to identify the factors including migration status associated with general health, physical health, and emotional health, respectively. Healthy Migrant Hypothesis was tested by comparing out-migrants with rural residents of the same origin. We compared the health status of returned migrants with that of migrants who stayed in destinations to examine the Salmon Bias Hypothesis. Moreover, we examined the change of health selectivity in 2012—2016. A second set of multivariate logistic regressions were applied to subsamples according to whether the respondents moved with family to further explore the effects of family migration.

Considering that physical health and emotional health might interact with each other through potential somatopsychic effects [41], physical health was controlled as an independent variable in emotional health analysis, and vice versa. General health was a relatively comprehensive health indicator. It was not controlled in physical health analysis because it already contained the influence of emotional health, and neither was in emotional health analysis. However, the physical health and emotional health were all controlled as independent variables in general health analysis, as they could affect overall health in different ways. The survey round variable was included in the regression models to adjust for the time effect on health. Considering the clustering of individuals at the household level, all analyses included random effects of households. Results were shown as average marginal effects (AMEs) of migration status. Each AME was extracted from the corresponding logistic regression model separately. The statistical significance level was defined as 0.05.

## 3. Results

### 3.1. Descriptive Analysis

Descriptive results of our analytic sample of 51,530 individuals are shown in Table 1. Rural-urban migrants accounted for 6.8% of the sample, while returned migrants were 8.2%. The percentages of urban and rural residents were 23.7% and 61.4%, respectively. Migrants were less likely to report poor health than the other three groups. An exception was that a higher percentage of migrants (16.5%) reported poor emotional health than urban residents (13.6%). Overall, returned migrants were the unhealthiest group.

**Figure 1:**
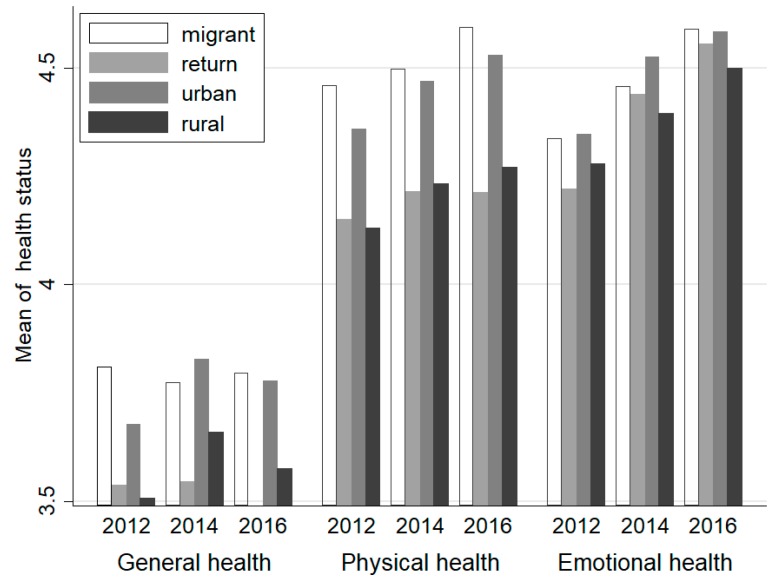
Health differences of four types of population in 2012–2016 (wave 1–3). Note: Health outcomes all transform into the five-point Likert Scale, with excellent health coded as 5 and poor health coded as 1.

We observed significant differences in the comparisons between different groups using the original self-rated health scores. The average health levels of four types of population are shown in Figure 1. Migrants show better general and physical health than rural residents in all three surveys, which adds support to the Healthy Migrant Hypothesis. Furthermore, results without controlling for other factors showed some preliminary support for the Salmon Bias Hypothesis: The health of returned migrants was worse than the migrants who stayed in destinations. All these differences were not significant in terms of emotional health. Considering that different characteristics among the four types, such as age, education, and gender, may affect the average level. These factors were also included in the multivariate analysis.

Specifically, migrants were the youngest group. The proportion of men was the highest among returned migrants (59.2%) compared with out-migrants, urban residents, and rural residents (47.1%, 47.3%, and 44.1%). Urban residents were the highest-educated group along with the highest self-rated socioeconomic scores. Out-migrants were more educated than rural residents and returned migrants, but their average self-rated socioeconomic score was the lowest (4.0). They worked longer with higher occupational hazard exposure rate (37.5%) than urban residents (21.7%), indicating adverse working conditions in the city. In terms of health behaviors, the proportion of current or former smokers was the highest among returned migrants (42.0%), followed by out-migrants (28.2%) and urban and rural residents (25.1% and 24.8%). Similarly, the proportion of drinkers was the highest among returned migrants (27.3%), followed by out-migrants (23.0%) and urban and rural residents (20.0% and 17.7%). Only 76.2% of out-migrants were covered by health insurance, lower than returnees, urban, and rural residents (93.1%, 83.2%, and 89.3%). But their residential communities were more well-equipped with fitness facilities (80.0%) and hospitals (2.6) than people living in the countryside. It indicated that rural infrastructure and health care resources were inferior to those of urban areas.

### 3.2. Healthy Migrant Hypothesis

Multivariate logistic regression results showed that migrants were generally and physically healthier than rural residents (AME = −0.27, *p* < 0.01; AME = −0.41, *p* < 0.001) (see Table 2), but they performed worse in terms of emotional health (AME = 0.20, *p* < 0.01). The absolute value of the average marginal effect of migration status increased over time. The differences between the years were statistically significant after carrying out LR tests for the models with and without interaction of year and migration status (*p* = 0.01; *p* < 0.01). This phenomenon suggested that the general and physical health gap between migrants and rural residents were widening. Moreover, migrants presented no health advantages but emotional health disadvantage comparing with urban residents (AME = 0.18, *p* < 0.01). 

Considering the effects of different health behaviors on the Healthy Migrant Effect, we also established models removing smoking and drinking from the control variables. The estimates are not shown but are generally the same as the results above. 

Migrants were divided into two groups according to whether to move with their families. Separate analyses of the two groups are reported in Table 3. Those who moved together with family members presented better general health and physical health than rural residents living with family (AME = 0.21, *p* < 0.01; AME = 0.35, *p* < 0.05), but the average marginal effects were reduced or became insignificant for the subgroup living alone. We assumed that family separation and lack of social support might lead to poor psychological health, while the average marginal effects in emotional health models were the same for both subgroups. 

### 3.3. Salmon Bias Hypothesis

In this analysis, we use the returned migrants as the reference group to test the Salmon Bias Hypothesis. Results are reported in Table 4.

Returned migrants experienced worse health in both general health (AME = 0.41, *p* < 0.001) and physical health (AME = 0.66, *p* < 0.001) when compared with migrants who remained in the destination. However, they were less likely to report poor emotional health than out-migrants (AME = −0.19, *p* < 0.05). The difference slightly varied in different years. We observed a widening gap between returned migrants and out-migrants as the absolute value of the average marginal effects increased by year. Both health gaps between migrants and rural residents and between migrants and returnees had widened over time, which suggested a possibly increasing trend of health selectivity.

Furthermore, the general health and physical health of returned migrants were even worse than their rural counterparts (AME = 0.14, *p* < 0.05; AME = 0.26, *p* < 0.001), as shown in Table 4. We also established models by grouping out-migrant and returned migrants together as ever-migrants (see Appendix A, Table A1), and found no more health advantages for them. They performed worse than urban residents (AME = 0.40, *p* < 0.001; AME = 0.24, *p* < 0.001) and showed no statistical difference when compared with rural residents.

To further explore health-related factors, we established regression models for the four types of population separately (see Appendix A, Table A2 for the model of returned migrants). Features such as being male, younger age, being married, having a higher education level, and having higher socioeconomic status were all associated with better health. The general health of returned migrants with the interprovincial migrant experience was significantly worse than those who migrated inside the province (OR = 0.70, *p* < 0.01). It suggested that the interprovincial migrants experienced a greater loss of health. More hospitals within the community and living in a community with fitness facilities played a positive role in improving rural and urban residents’ health but had no effect in the models of returned migrants and out-migrants. It might be because migrants did not make full use of the health resources of their communities. For health behaviors, smoking and drinking were associated with better health. It might be because unhealthy people were more likely to avoid unhealthy behaviors for health reasons. Occupational hazard exposure was negatively associated with the health of all types of population. 

## 4. Discussion

Most of the previous studies were focused on international or internal migrants in developed countries, and only a few studies were conducted in developing countries. This study investigated the relationship between health and internal migration in developing countries by examining empirical evidence for the selectively healthy migrants in the context of an unprecedented rural-urban migration in China. Our analysis can provide support for the Healthy Migrant Hypothesis that migrants have general health and physical health advantages over rural residents. However, we find that migrants have worst emotional health among the four groups, indicating that they might be physically healthy but mentally ill [42]. Our findings are different from previous research. Although the previous research used CLDS 2012 survey data [36], it only classified the answer “none/few” as “poor emotional health,” while “sometimes” indicated “good health” improperly. Thus, in the previous study, the percentage of “poor emotional health” was too low (3.69%) after the transformation. Besides, existing studies on migrants’ psychological health yield mixed conclusions, some of which also find severe psychological problems of migrants are due to discrimination, pressure and poor social capital [27,43,44]. Our research provided supportive evidence for the conclusion. 

Our study also finds that migrants do not show better health than urban residents. This finding differs from previous studies conducted in metropolitan areas such as Beijing [27], Hangzhou [45], and Shanghai [46]. Migrants might be more positively selected on health than those in smaller cities. The weakened health selection might be because the CLDS covered large, medium, and small cities across the country. Also, the original rural-urban health gap [47] might also lead to a lack of health advantages for migrants. 

The Salmon Bias Hypothesis is validated in this study. The health differences between out-migrants and returnees suggest that poor general and physical health might be one of the reasons for returning home, but returnees’ better emotional health than migrants suggests a possible positive effect of returning on emotional health. Ever-migrants no longer have any health advantages, which demonstrates that ignoring selective return migration might upwardly bias migrants’ health. Yet, returning to rural areas with worse health services and living conditions could be another reason for ill health. Returnees’ health is not only worse than out-migrants, but also worse than rural residents who never migrated. As unhealthy people return to the countryside, they bring a greater health burden and higher demands than the underdeveloped rural medical care system could satisfy. 

We expect that the pattern of health selection could be less intense under the circumstance of improvements of social services and diminishing stringent requirements for migrants. In contrast, a possibly increasing trend of health selectivity is found in this paper as the health gaps are widening. It suggests that there are increasing obstacles for migrants to overcome. Good general and physical health are essential to settling down in cities. Further, increasing family migration could be the reason for increasing selectivity. The 2016 National Survey Data of the Dynamic Monitoring of the Migrant Population shows that nearly 60% of the new generation of married migrants migrate with their spouses and children, and more and more families are also beginning to take the elderly with them [20]. Migration decisions are made not only for individuals, but also for the whole family [48]. With the trend of migrating with family, health selection is increasingly intense for migrants aged 15 to 64 years in this paper. It might be because bringing family to the destination requires better general and physical health to overcome more obstacles. We find no evidence for the improvement of the emotional health of family migrants.

The relationship between migration and health investigated in this paper has strong policy implications. For migrants in cities, firstly, the poor emotional health of migrants deserves more attention and intervention. Second, as more and more people migrate with spouses, children, and the elderly, their needs for public services and social security increasingly expand. The government should also increase the provision of welfare and public resources for their family members. Third, since migrants are exposed to occupational hazards with a much higher percentage, it is necessary to improve workplace safety and strengthen occupational protection. 

For returned migrants, the unhealthy returnees burden the rural areas, which would broaden the gap between urban and rural health. The increasing trend of health selectivity will exacerbate this health inequality. In essence, the rural areas are exporting good health and reimporting ill-health [42]. The government must promote the New Rural Cooperative Medical System and increase the reimbursement rate. Therefore, it is urgent to establish and improve the primary healthcare service systems in rural areas in current circumstances.

## 5. Limitations

A few limitations should be noticed. First, we only analyzed three self-reported health outcomes, which were recorded at all three rounds of the survey. Migrants’ health advantages might be overestimated, as they are more likely to ignore and under-report poor physical health [45], though perceived health is shown to be related to other actual health outcomes [49,50]. More health indicators, especially objective ones, should be addressed in future studies. Second, our research only focused on the labor force that was more likely to be job-related migrants. Information on the purpose of migration is unavailable to further analyze the health selectivity under different migration patterns. Third, our study is cross-sectional, and we cannot infer the causal relationship between health and migration. It can only give us hints of the relationship between the two. We acknowledge that the unhealthy assimilation of previous immigrants might influence the results of health comparisons, but some studies revealed that migrants’ initial health advantage remained stable [51,52] comparing with native residents. We believe that the increased health disparities are more likely to be the result of increasing selectivity as assimilation does not lead to an increased health advantage. However, the impact of migration on health might warrant a future longitudinal study. 

## 6. Conclusions

This paper examined empirical evidence for two processes regarding health of migrants in China: The Healthy Migrant Hypothesis, which holds that rural-urban migrants are healthier than non-migrants in rural areas, and the Salmon Bias Hypothesis, which posits that migrants still outside are healthier than returned migrants. In contrast to the results of general and physical health, migrants have the worst emotional health, which deserves more attention and intervention. Similarly, it requires better general and physical health, but not emotional health, to bring family to the destination. 

The widening general and physical health gaps between migrants, rural residents, and returnees imply a possibly increasing trend of health selectivity. There are increasing number of obstacles for migrants to overcome, and family migration’s high requirement for health might contribute to it. It is urgent to establish and improve the primary health care service systems in rural areas in current circumstances. This paper may deepen the understanding of the relationship between health and internal migration in other developing countries. The study of health selectivity should consider not only the change of policy environment, but also the needs and characteristics of migrants in the new era.

## Figures and Tables

**Table 1:** Percentages and means of variables by migration status: China Labor-force Dynamics Survey (CLDS) 2012–2016.

Variables	Total	Migrants	Return	Urban	Rural
Self-reported poor general health * (%)	13.4	7.0	15.9	7.7	16.0
Self-reported poor physical health * (%)	22.3	14.5	27.2	15.8	25.1
Self-reported poor emotional health * (%)	16.7	16.5	18.4	13.6	17.7
Male * (%)	46.3	47.1	59.2	47.3	44.1
Age *	43.1	36.4	42.6	43.1	43.9
Married * (%)	82.1	81.1	90.0	78.4	82.5
Education * (%)					
Primary or lower	37.7	30.4	44.6	10.0	48.2
Junior	33.1	40.7	42.5	25.8	33.8
Senior	16.1	16.7	10.2	28.4	12.0
College or above	13.1	12.2	2.7	35.8	6.0
Self-rated SES * (scale 1-10)	4.4	4.0	4.1	4.6	4.3
Workweek * (hour)	30.7	43.9	48.5	25.8	29.8
Occupational hazards exposure * (%)	23.1	37.5	31.1	21.7	20.2
Having smoking history * (%)	26.5	28.2	42.0	25.1	24.8
Having drinking history * (%)	19.4	23.0	27.3	20.0	17.7
Having health insurance * (%)	87.3	76.2	93.1	83.2	89.3
Living alone * (%)	3.9	9.6	2.9	4.8	3.1
Having fitness facilities in residential community * (%)	62.3	80.0	52.2	77.1	56.0
Number of hospitals in residential community *	2.3	2.6	1.6	3.2	2.0
*N*	51,530	3511	4200	12,183	31,636

Notes: * *p* < 0.05. *p*-value is obtained from chi-square tests or one-way analysis of variance, depending on whether the variable is categorical or continuous.

**Table 2:** Marginal effects of migration status (comparing with migrants).

	General Health	Physical Health	Emotional Health
Overall sample			
Migration status (Ref: migrants)			
Returned migrants	−0.41 ***(−0.62, −0.20)	−0.66 ***(−0.81, −0.51)	0.19 *(0.04, 0.34)
Urban residents	0.12(−0.08, 0.33)	−0.16(−0.31, 0.02)	0.18 **(0.05, 0.32)
Rural residents	−0.27 **(−0.45, −0.09)	−0.41 ***(−0.53, −0.27)	0.20 **(0.07, 0.32)
2012			
Migration status (Ref: migrants)			
Returned migrants	0.04(−0.36, 0.45)	−0.45 **(−0.72, −0.17)	−0.08(−0.34, 0.18)
Urban residents	0.19(−0.20, 0.58)	−0.26(−0.52, 0.01)	0.13(−0.11, 0.37)
Rural residents	−0.07(−0.42, 0.29)	−0.25 **(−0.46, −0.04)	0.08(−0.14, 0.30)
2014			
Migration status (Ref: migrants)			
Returned migrants	−0.58 **(−0.95, −0.22)	−0.55 ***(−0.81, −0.30)	0.30 *(0.03, 0.57)
Urban residents	0.04(−0.30, 0.39)	−0.07(−0.31, 0.16)	0.37 **(0.13, 0.60)
Rural residents	−0.24 (−0.56, 0.07)	−0.32 **(−0.57, −0.08)	0.28 **(0.07, 0.49)
2016			
Migration status (Ref: migrants)			
Returned migrants	−0.64 ***(−0.98, −0.29)	−0.92 ***(−1.18, −0.67)	0.39 **(0.11, 0.68)
Urban residents	0.08(−0.26, 0.42)	−0.22(−0.47, 0.02)	0.22(−0.04, 0.47)
Rural residents	−0.42 **(−0.73, −0.12)	−0.57 **(−0.79, −0.35)	0.21(−0.02, 0.44)

Note: Average marginal effects are from logistic regression models. 95% confidence intervals are shown in the brackets. Model adjustments include gender, age, marital status, education level, self-rated SES, workweek, smoking history, drinking history, health insurance coverage, whether living alone, whether having fitness facilities in residential community, number of hospitals in residential community, and other health outcomes. *** *p* < 0.001, ** *p* < 0.01, * *p* < 0.05.

**Table 3:** Marginal effects of migration status by whether living alone.

	General Health	Physical Health	Emotional Health
Overall sample	35,147		
Dichotomous comparison (migrants vs. rural residents)	0.23 ***(0.05, 0.42)	0.37 *(0.23, 0.50)	−0.22 **(−0.35, −0.09)
By whether living alone			
Yes (vs. rural residents)	1.16(−0.31, 2.63)	0.07 *(0.01, 0.14)	−0.22 *(−0.41, −0.04)
N	1321		
No (vs. rural residents)	0.21 **(0.02, 0.40)	0.35 *(0.21, 0.47)	−0.22 **(−0.35, −0.09)
*N*	33,826		

Note: Average marginal effects are from logistic regression models. 95% confidence intervals are shown in the brackets. Model adjustments are the same as Table 2. *** *p* < 0.001, ** *p* < 0.01, * *p* < 0.05.

**Table 4:** Marginal effects of migration status (comparing with returned migrants).

	General Health	Physical Health	Emotional Health
Overall sample			
Migration status (Ref: Return)			
Out-migrants	0.41 ***(0.20, 0.62)	0.66 ***(0.51, 0.81)	−0.19 *(−0.34, −0.04)
Urban residents	0.54 ***(0.38, 0.70)	0.50 ***(0.38, 0.62)	−0.01(−0.14, 0.12)
Rural residents	0.14 *(0.02, 0.27)	0.26 ***(0.16, 0.35)	0.01(−0.10, 0.11)
2012			
Migration status (Ref: Return)			
Out-migrants	−0.04(−0.45, 0.36)	0.45 **(0.17, 0.72)	0.08(−0.18, 0.34)
Urban residents	0.15(−0.17, 0.46)	0.19 (−0.03, 0.41)	0.21(−0.01, 043)
Rural residents	−0.11(−0.35, 0.13)	0.12 (−0.05, 0.29)	0.16(−0.01, 0.33)
2014			
Migration status (Ref: Return)			
Out-migrants	0.58 **(0.22, 0.95)	0.55 ***(0.30, 0.81)	−0.30 *(−0.57, −0.03)
Urban residents	0.63 ***(0.35, 0.90)	0.48 ***(0.27, 0.69)	0.07(−0.16, 0.30)
Rural residents	0.34 **(0.12, 0.56)	0.30 ***(0.13, 0.48)	−0.02(−0.21, 0.18)
2016			
Migration status (Ref: Return)			
Out-migrants	0.64 ***(0.29, 0.98)	0.93 ***(0.67, 1.18)	−0.39 **(−0.68, −0.11)
Urban residents	0.72 ***(0.45, 0.98)	0.70 ***(0.50, 0.90)	−0.18(−0.42, 0.07)
Rural residents	0.21 *(0.01, 0.41)	0.35 ***(0.19, 0.51)	−0.18(−0.38, 0.02)

Note: Average marginal effects are from logistic regression models. 95% confidence intervals are shown in the brackets. Model adjustments are the same as Table 2. *** *p* < 0.001, ** *p* < 0.01, * *p* < 0.05.

## Appendix A

**Table A1:** Marginal effects of migration status (comparing with ever-migrants).

	General Health	Physical Health	Emotional Health
Overall sample			
Migration status (Ref: ever-migrants)			
Urban residents	0.40 ***(0.25,0.55)	0.24 ***(0.13,0.35)	0.08(−0.03,0.19)
Rural residents	0.01(−0.09,0.12)	0.02(−0.06,0.10)	0.08(−0.00,0.17)
2012			
Migration status (Ref: ever-migrants)			
Urban residents	0.16(−0.12,0.44)	0.02(−0.17,0.21)	0.17(−0.01,0.36)
Rural residents	−0.10(−0.30,0.11)	−0.02(−0.17,0.13)	0.13(−0.01,0.28)
2014			
Migration status (Ref: ever-migrants)			
Urban residents	0.41 **(0.17,0.66)	0.25 **(0.07,0.42)	0.21 **(0.02,0.40)
Rural residents	0.14 (−0.05,0.33)	0.08(−0.06,0.22)	0.12(−0.03,0.27)
2016			
Migration status (Ref: ever-migrants)			
Urban residents	0.51 ***(0.27,0.75)	0.35 ***(0.17,0.53)	−0.00(−0.21,0.20)
Rural residents	0.01(−0.16,0.19)	0.02(−0.11,0.16)	−0.02(−0.18,0.14)

Note: Average marginal effects are from logistic regression models. 95% confidence intervals are shown in the brackets. Model adjustments are the same as Table 2. *** *p* <0.001, ** *p* <0.01, * *p* <0.05.

**Table A2:** Relevant factors affecting health of returned migrants.

	General Health	Physical Health	Emotional Health
Physical health (Ref: fair/poor)	11.05 ***(7.88,15.48)	-	8.95 ***(6.96,11.51)
Emotional health (Ref: fair/poor)	1.63 ***(1.24,2,13)	11.91 ***(8.77,16.17)	-
Flow distance ^1^ (Ref: intra-provincial)	0.70 **(0.56,0.89)	0.85(0.71,1.02)	1.06(0.88,1.27)
Gender (Ref: female)	0.97(0.68,1.34)	0.90(0.69,1.17)	1.71 ***(1.29,2.26)
Age	0.93 ***(0.92,0.94)	0.95 ***(0.94,0.96)	1.01(1.00,1.02)
Marital status (Ref: single/divorced)	1.37(0.89,2.09)	0.96(0.68,1.35)	0.99(0.71,1.38)
Education (Ref: primary or lower)			
Junior	1.78 ***(1.437,2.30)	1.66 ***(1.35,2.04)	1.07(0.87,1.31)
Senior	1.66 *(1.07,2.58)	2.18 ***(1.54,3.08)	0.95(0.68,1.31)
College or above	2.10(0.65,6.75)	3.42 **(1.58,7.41)	0.72(0.40,1.30)
Socioeconomic status	1.15 ***(1.810,1.23)	1.13 **(1.07,1.19)	1.06 *(1.01,1.12)
Workweek (Ref: ≤44h)			
>44h	1.10(0.85,1.42)	0.99(0.80,1.21)	1.09(0.88,1.35)
0h	0.57 ***(0.41,0.78)	0.65 **(0.50,0.84)	0.79(0.61,1.02)
Smoking (Ref: not smoking)	1.21(0.89,1.66)	1.35 *(1.04,1.74)	0.72 *(0.55,0.94)
Drinking (Ref: not drinking)	1.40 *(1.04,1.87)	1.53 ***(1.21,1.94)	0.95(0.75,1.21)
Health insurance (Ref: without)	1.07(0.69,1.66)	0.86(0.60,1.23)	1.07(0.76,1.53)
Living alone (Ref: with family)	0.57(0.31,1.05)	0.81(0.48,1.38)	0.81(0.4821,1.36)
Fitness facilities (Ref: without)	1.23(0.97,1.55)	1.07(0.89,1.30)	1.02(0.85,1.23)
Number of hospitals in community	0.98(0.91,1.06)	1.01(0.95,1.07)	1.02(0.96,1.08)
Occupational hazards (Ref: not exposed)	1.21(0.91,1.60)	0.72 **(0.58,0.89)	1.04(0.84,1.30)
Wave (Ref: 1)			
2	0.64 ***(0.47,0.86)	0.77 *(0.60,0.98)	1.43 **(1.14,1.79)
3	0.61 **(0.45,0.82)	0.67*(0.53,0.86)	1.92 ***(1.51,2.44)

Note: Adjusted odds ratios and 95% confidence intervals are shown. ^1^ “Flow distance” was added only in the model of returned population. *** *p* < 0.001, ** *p* < 0.01, * *p* < 0.05.
